# FeOx-TiO_2_ Film with Different Microstructures Leading to Femtosecond Transients with Different Properties: Biological Implications under Visible Light

**DOI:** 10.1038/srep30113

**Published:** 2016-07-22

**Authors:** Sami Rtimi, Cesar Pulgarin, Victor A. Nadtochenko, Fedor E. Gostev, Ivan V. Shelaev, John Kiwi

**Affiliations:** 1 Ecole Polytechnique Fédérale de Lausanne, EPFL-SB-ISIC-GPAO, Station 6, CH-1015 Lausanne, Switzerland; 2 N. N. Semenov Institute of Chemical Physics, Russian Academy of Sciences, str. Kosygina 4, 119991 Moscow, Russia; 3 Moscow State University, Department of Chemistry, Leninskiye Gory 1-3, 119991, Moscow, Russian Federation, Russia

## Abstract

This study presents the first report addressing the effect of FeOx-TiO_2_ films microstructure on the transients detected by fast spectroscopy related to the long-range bacterial inactivation performance. The different fast kinetic femtosecond transient spectroscopy is reported for each FeOx+TiO_2_ microstructure. The lifetime of the short transient-species and the oxidative intermediate radicals generated under light were identified. Co-sputtered FeOx-TiO_2_ on polyethylene films presenting random distribution for both oxides were compared with sequentially sputtered FeOx/TiO_2_ films made up only by FeOx in the topmost layers. The ratio FeOx:TiO_2_ was optimized to attain the highest photo-conversion. By X-ray fluorescence, the Fe:Ti ration was found to be ~1.4 in the film bulk and by XPS-etching a ratio of 4:1 was found on the photocatalyst top-most layers. For co-sputtered FeOx-TiO_2_-PE films, the FeOx-TiO_2_ heterojunction led to electron injection from the FeOx to lower-lying TiO_2_ trapping states. The film optical properties, particle size, roughness, hydrophobic-hydrophilic shift and temporal evolution of the transient *redox* states were characterized in detail. Films with different microstructure led to different antibacterial activity. This suggests that the FeOx-TiO_2_-PE microstructure and not the position of the potential energy level of the semiconductors FeOx and TiO_2_ control the charge transfer under light irradiation.

Iron-oxide with about 2.1–2.2 eV present an ideal band-gap for water splitting^1^,^2^, absorbs sunlight in the 500–600 nm range and show Fe-surface plasmon resonance wavelength at λ = 590 nm. Hematite is an inexpensive and readily available oxide on the earth surface. Binary-oxides semiconductors due to their optical absorption and semiconductor behavior have been widely used for environmental decontamination purposes like FeOx-TiO_2_. These binary oxides play an important role in pollutant and bacterial abatement involving redox processes^3^,^4^. The mineral deposits of ilmenite (FeTiO_3_) leach out Fe/Fe-ions and to a lesser degree Ti important in natural cleaning cycles. After interacting with organic matter in water bodies, the Ti and Fe-species/ions re-crystallize again on the original matrix after catalyzing the abatement of pollutants in the ppb to the micromolar range^3^,^4^.

An emerging area in environmental research is the study of solid-phase redox reactions by ultrafast time resolved spectroscopy. Femto-second transient absorption (TA) on FeOx-TiO_2_ films, relaxation dynamics and charge-transfer has been partially reported^5^,^6^,^7^,^8^,^9^,^10^. Studies report the absorbance of TiO_2_ in dye-sensitized cells. These studies shoeing modified TiO_2_ absorbing light in the visible region have been reported over the last two decades addressing the fate of the excited transients leading to charge separation^11^,^12^,^13^,^14^.

We address in this study the FeOx-TiO_2_-PE transient kinetics using a femto-second pump (25 fs) at various wavelengths in the visible region. The transient lifetime/absorption will be monitored on the co-sputtered and sequentially sputtered films. Also, the characterization of the film microstructure, layer composition, optical properties, particle size, surface roughness, contact angle and surface redox species will be reported in detail. The charge transfer for films will be suggested for films with a different microstructure based on the data obtained during the course of this work. The present study is a continuation of our recent work on co-sputtered Fe/Ti photocatalysts^15^. The charge migration and photo-induced charge separation is reported on the sputtered, uniform, adhesive, stable and reproducible films.

## Results and Discussion

### Optical Absorption of FeOx-TiO_2_-PE Films

**Figure 1:**
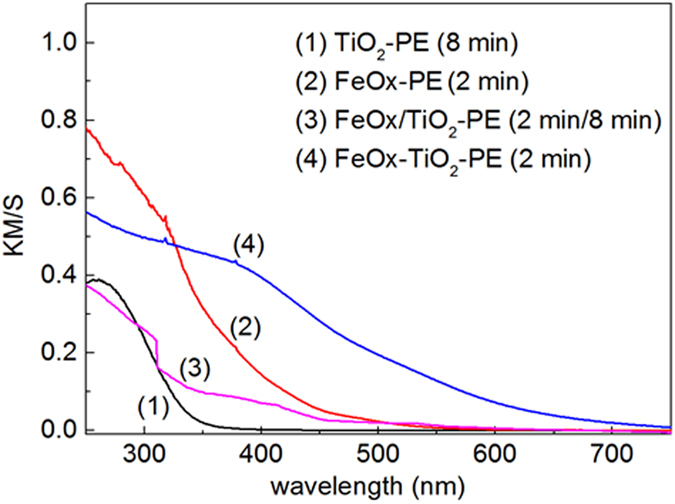
Diffuse reflection spectroscopy (DRS) showing the Fe(III) shifting band gap excitation of TiO_2_ to visible region in the samples: (1) TiO_2_-PE (8 min) (2) FeOx-PE, (2 min) (3) sequentially sputtered FeOx/TiO_2_-PE (2 min FeOx/8 min TiO_2_) and (4) co-sputtered FeOx-TiO_2_-PE for (2 min).

Figure 1 shows the DRS spectra in Kubelka-Munk units for films of: TiO_2_-PE, FeOx-PE, sequentially sputtered FeOx/TiO_2_-PE and co-sputtered FeOx-TiO_2_-PE. The sputtering times for Fe(III) noted in the caption of Fig. 1, have been optimized to find the most suitable ratio Fe(III) : TiO_2_ to photo-catalyze the bacterial inactivation kinetics. The FeOx-TiO_2_-PE films displayed absorption in the visible region >400 nm, due to the charge transfer from TiO_2_ to Fe(III) layers^15^,^16^,^17^.

The light absorption between 400 nm and 500 nm in Fig. 1, is attributed to the interfacial charge transfer (IFCT) between TiO_2_ and Fe(III) and the weak absorption >500 nm to the short lived Fe d-d inter-band transitions. Fe(III) introduces intra-gap energy states in TiO_2_ in the co-sputtered and sequentially sputtered samples. During the sputtering of FeOx-TiO_2_ on PE, TiO_2_ loss of oxygen leads to oxygen vacancies. The electron-pair deficient oxygen vacancy and the electrons left in the TiO_2_ network react with Ti^4+^-ions to form Ti^3+^ centers. These localized states (intra-gap states) allow the incident light to induce reactions in the sub band-gap domain^18^,^19^. Serpone *et al.*^20^, reported that the formation of oxygen vacancies involves TiO_2_ reduction associated with the TiO_2_ red shift. The Fe(III) incorporated into the TiO_2_ lattice substitutes Ti^4+^ inducing oxygen vacancies to conserve the network electro-neutrality. The amount of the vacancies has been reported to be one-half of the Fe(III) found in the network Ti^4+ ^11,12,13,14.

### Femto-second transients generated on FeOx-TiO_2_ and FeOx/TiO_2_ films

**Figure 2:**
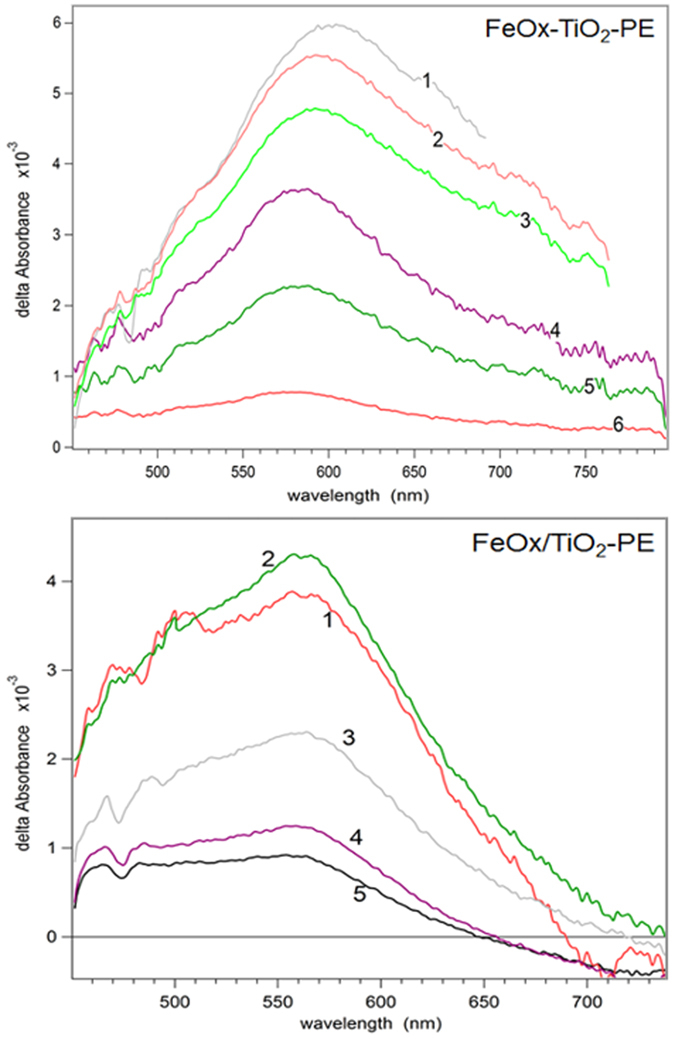
Transient spectra of the co-sputtered FeOx-TiO_2_-PE and FeOx/TiO_2_-PE films as a function of wavelength induced by femto-second pulse excitation at 425 nm, with pulses. Time delays for FeOx-TiO_2_-PE film: (1) 100 fs; (2) 160; (3) 300 fs; (4) 2.3 ps; (5) 16 ps; (6) 500 ps. Time delays for FeOx/TiO_2_-PE film: (1) 100 fs; (2) 150 fs; (3) 1.8 ps; (4) 17 ps; (5) 500 ps.

Figure 2 shows the transient spectra of the co-sputtered (FeOx-TiO_2_-PE) film and for the sequentially sputtered (FeOx/TiO_2_-PE) film induced by femto-second laser pulses at 425 nm (100 nJ/40 fs). For the FeOx-TiO_2_-PE co-sputtered films, the transient absorption spectra *delta* were observed with Absorbance >0 with maximum ~580–600 nm. For the sequentially sputtered FeOx/TiO_2_-PE films, the excited state absorption (ESA) transient band *delta* Absorbance >0 with maximum close to 570–580 nm were observed and bleaching bands (*delta* Absorbance <0) were detected for wavelength longer than 650 nm. The bleaching bands were due to the (a) depletion of the ground state population, (b) filling of the higher energy states corresponding to the optical transitions or (c) due to Kerr shift of the edge absorption band. The excited state absorption (ESA) induced by laser spectroscopy reported in Fig. 2 was due to the overlapping of the TiO_2_ cb(e^−^) with the FeOx vb(h^+^) bands and has been reported for some transition metals^21^. The ESA bands presented a spectrum close to hematite. This agrees with the spectra of the co-sputtered films shown in Fig. 2a, trace 1. Figure 2 shows a FeOx maximum absorption at 570 nm for the topmost layers in the sequentially sputtered film (FeOx/TiO_2_-PE). The co-sputtered films in Fig. 2 show a maximum at 610 nm for the FeOx. The FeOx red shift in the co-sputtered TiO_2_-FeOx-PE film originates from the fact that only the FeOx layers absorb the light in the later film.

In Fig. 2, a prominent absorption band is detected for the sequentially sputtered film (FeOx/TiO_2_-PE) but it is not observed for the co-sputtered FeOx-TiO_2_-PE film. The wide absorption transient band delta Absorbance >0 between 430 nm and 650 nm in Fig. 2 can be attributed to local excitons (d-d excited Fe-ions) or to the charge carrier absorption in FeOx^22^. The ESA band shapes have been reported with a similar optical absorption as found during the preparation of iron-oxide colloids. Qualitatively transient absorption data for FeOx-TiO_2_-PE and FeOx/TiO_2_-PE were similar (Fig. 2). The main difference is the bleaching observed in the FeOx/TiO_2_-PE film due to the depletion of the ground state (g.s.) population of FeOx. The observed depletion may involve: (a) electron injection from FeOx into the TiO_2_ low lying trapping-states positioned between −0, 2 and up to −1.0 eV below the TiO_2_ cb (−0.1 eV)^23^,^24^,^25^,^26^ and (b) the Fe(II) oxidation in the FeOx oxidation. The real position of the TiO_2_ trapping-sates as noted in point a) is controversial since it has been measured/assigned by different techniques^23^,^24^,^25^,^26^. The role of the intra-gap Fe-states in TiO_2_ cannot be discounted, but no quantitative information about the intra-gap potential energy levels is available at this time.

**Figure 3:**
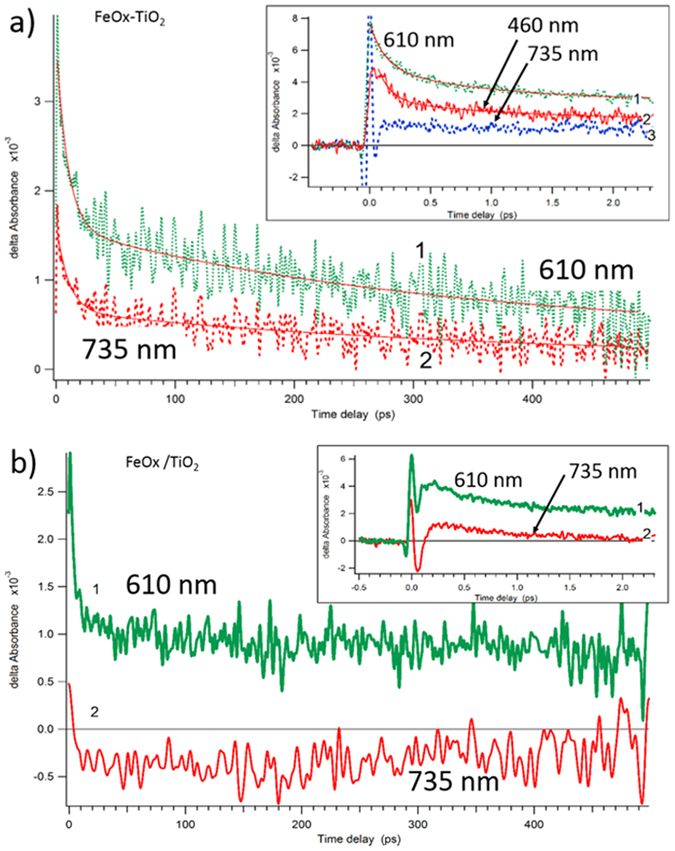
(**a**) Transient absorption decay profiles of the co-sputtered FeOx-TiO_2_-PE films for probe wavelengths at 610 nm and 735 nm. Time delays of 450 ps and of 2 ps in the insert. (**b**) Transient absorption decay profiles of FeOx/TiO_2_-PE films for probe wavelengths: 610 nm and 735 nm. Time delays of 450 ps and of 2 ps in the insert.

Figure 3 shows the spectra decay of the sequentially sputtered films absorbing light through the FeOx-layers with an initial time delay of 0.146 ps compared to the co-sputtered films with an initial time delay of 100 fs. Interestingly enough, Fig. 3 shows that the spectral decay was very similar, within 16–18 ps. In a recent study^15^ we did not provide unambiguous identification for the FeOx or TiO_2_ in the co-sputtered FeOx-TiO_2_-PE film. In Fig. 3 transients do not show exponential decay within the time ranges: a) 0–2 ps and b) 0–00 ps. A satisfactory fitting for the transient decay could only be achieved in the restricted time window. The FeOx-TiO_2_-PE and FeOx/TiO_2_-PE films present a fast decay component with inverted time constant close to 2.4–3.5 ps^−1^ for the FeOx/TiO_2_-PE film and of 1.3–1.7 ps^−1^ for FeOx-TiO_2_-PE film. Up-to 500 ps, the transient decay for both films could be fitted by a double-exponential decay. Non-exponential decays have been reported due to the dispersive charge recombination or exciton-decay presenting a high degree of charge distribution disorder^26^,^27^. The non-exponential decay in Fig. 3a up to 500 ps were fitted by Kohlrausch’s stretched exponential fitting^28^ for the FeOx-TiO_2_-PE film (for the fitting details see Supplementary Material S1). For the FeOx/TiO_2_-PE films, Fig. 3b shows the FeOx transient absorption maximum at 610 nm reaching 735 nm, decaying within 100 ps (for details see Supplementary Material S2)^29^,^30^.

**Figure 4:**
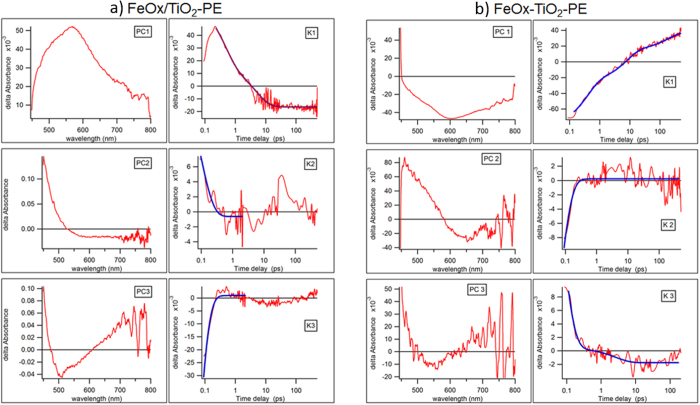
(**a**) SVD analysis of the femto-second transient spectra absorption for FeOx-TiO_2_-PE films within 100 ps. The U(λ) are shown in left axes (PC). Time-dependent profiles V^T^(t) are shown in the right axes (K). (**b**). SVD analysis of the femto-second transient spectra absorption for FeOx/TiO_2_-PE films within 100 ps. The U(λ) are shown in left axes (PC). Time-dependent profiles V^T^(t) are shown in the right axes (K).

Figure 4 shows in the first column U(λ) and V^T^ matrixes. U(λ) present the linear composition of the spectra intermediates and columns V^T^(t) present the linear composition of the kinetic traces determined by factor analysis described previously^31^. The results of the multi-exponential deconvolution of the time-dependent profiles V(t) are summarized in Table 1. Both FeOx-TiO_2_-PE and FeOx/TiO_2_-PE films show fast kinetics between 50 and 100 fs followed by a more moderate kinetics up-to 500 fs. The FeOx-TiO_2_-PE film presents a slow kinetic component with a decay time of ~185 ps, while the FeOx/TiO_2_-PE film presents a slow kinetic component of ~5 ps. The single decays for both film transients are shown in Supplementary Material S3.

Factor analysis methods, such as SVD^31^ are applied to gain insight on the complexity of the film decay kinetics. These methods have been used to model the transient decay reported in Fig. 4 (for more details see Supplementary Material 4).

It has been reported by Gilbert *et al.*^32^ that FeOx transient absorption (TA) shows small signal persisting after 200 ps between 460–700 nm following photo-excitation at 520 nm. In our case, the tail of long lived species (remaining after 500 ps) are 16% and 11% for FeOx-TiO_2_ and FeOx/TiO_2_.

**Figure 5:**
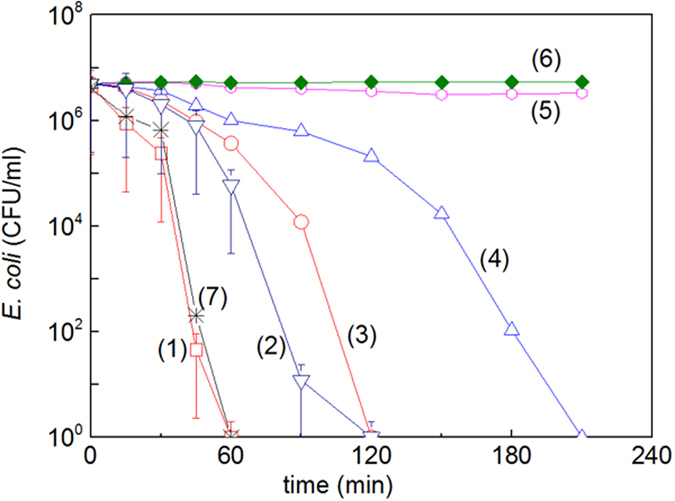
*E. coli* inactivation kinetics on: (1) FeOx-TiO_2_ co-sputtered for 2 min under sunlight simulated irradiation (52 mW/cm^2^) (2) FeOx/TiO_2_ sequentially sputtered for 8 min with TiO_2_ and subsequently by 2 min FeOx, (3) FeOx-PE sputtered for 2 min, (4) TiO_2_-PE sputtered for 8 min, (5) PE under light irradiation and (6) FeOx-TiO_2_-PE co-sputtered for 2 min in the dark. (7) FeOx-TiO_2_ co-sputtered for 2 min, irradiated under Suntest simulated (52 mW/cm^2^) light in the presence of a cut-off filter 400 nm.

The distinct transients generated in the FeOx/TiO_2_-PE film and FeOx-TiO_2_-PE co-sputtered film led to different bacterial inactivation kinetics as shown next in Fig. 5.

### Bacterial inactivation, oxidative radicals scavenging and changes induced in the film hydrophobicity under light

Figure 5, (trace 1) shows the bacterial inactivation within 60 min for FeOx-TiO_2_-PE films sputtered for 2 min. Figure 5 (trace 2) shows the bacterial inactivation for FeOx/TiO_2_-PE films sputtered for 8 min with TiO_2_ followed by 2 min with FeOx. Figure 5, traces 3 and 4 show the inactivation for FeOx-PE and TiO_2_-PE films. Control experiments show that bacterial inactivation does not proceed on PE under sunlight in Fig. 5, trace 5 and in the dark in trace 6. The FeOx-TiO_2_-PE films irradiated in the presence of a cut-off filter 400 nm, led to the same bacterial inactivation kinetics as in the absence of the cut-off filter. This proofs that the incoming light was absorbed only by the FeOx specoes in the co-sputtered films.

The bacterial reduction for an initial loading of 3.9 10^6^ (CFU/ml) by addition of the (2 mM) EDTA-Na a hole vb(h^+^) scavenger lead to a reduction of ~60%. Adding DMSO (2 mM), an OH-radical scavenger a bacterial reduction of about 2-orders of magnitude was observed after one hour on co-sputtered films and only of one order of magnitude in the case of the sequentially sputtered films. SOD added at pH 6, scavenged the O_2_°^−^-radicals 3 times more effectively on co-sputtered films compared to sequentially sputtered films. Co-sputtered films were more effective in the production of highly oxidative radicals leading to bacterial inactivation and these results are consistent with the data reported in Fig. 5, where the bacterial inactivation is shown to proceed faster on photo-activated co-sputtered films.

By contact angle experiments (CA), the initial hydrophobic angle found for co-sputtered films were ~96°. Within 60 min irradiation, the CA decreases to 11° implying a significant transformation to the hydrophilic region. In the case of the sequentially sputtered films, the initial CA-angle decreases from 103° to 25° within 120 min. The transformation from hydrophobic to hydrophilic surfaces occurred within the time of bacterial inactivation on both films under light irradiation.

**Figure 6:**
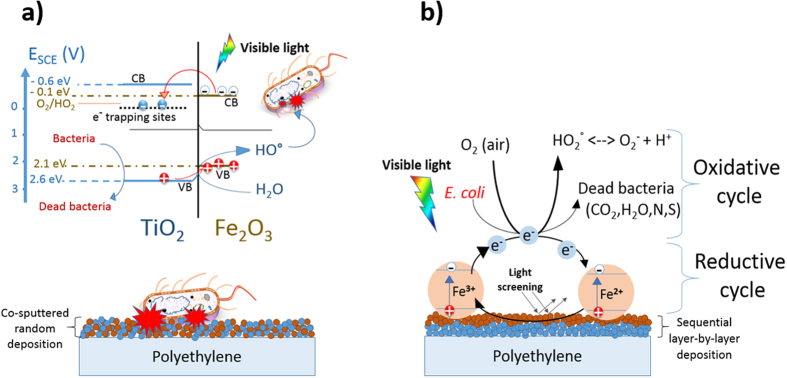
(**a**) Simplified mechanism for bacterial inactivation by co-sputtered by FeOx-TiO_2_-PE films under visible light. (**b**) Mechanism suggested for bacterial inactivation mediated by sequentially sputtered FeOx/TiO_2_-PE films under visible light.

A mechanistic scheme for the intervention of co-sputtered FeOx-TiO_2_-PE films leading to bacterial inactivation is shown in Fig. 6a. A heterojunction in the FeOx-TiO_2_-PE film leads to charge separation, charge transport and finally quasi-Fermi level equilibration at the FeOx:TiO_2_ interface. The FeOx electron transfer to low-lying TiO_2_ trapping-states is shown in Fig. 6a. The co-sputtered FeO_X_-TiO_2_-PE films led to a higher bacterial inactivation, since a decrease in the FeOx electron-hole recombination rate occurs due to the e-injection into TiO_2_. Some steps involving the transfer of Fe_2_O_3_ cb(e-) into the TiO_2_ trapping-sites are suggested below:


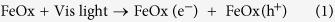



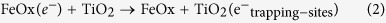



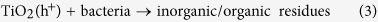


Figure 6b suggests for sequentially sputtered FeOx/TiO_2_-PE film an epitaxial growth of FeOx-species takes place on the already sputtered TiO_2_ under-layers^33^,^34^. In this case the mechanism is suggested in eq.(4) leading to the short lived unstable bacteria cation (+)


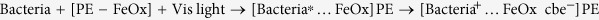


The sequentially FeO_X_-TiO_2_/PE film as shown in Fig. 5, induce bacterial inactivation kinetics similar to the one induced by FeOx-PE. This is due to three factors: (a) the high concentration of Fe in the FeOx layers (see Table 2)^12^,^13^,^14^,^15^,^16^,^22^,^35^,^36^, (b) the Fe_2_O_3_ very rapid e^−^/h^+^ recombination preventing its use as a photocatalyst as reported by several studies^9^,^10^,^25^,^26^,^27^ and (c) the FeOx are the only species in the layers exposed to light and additional channels compared with the co-sputtered films probably intervene in the bacterial inactivation. In the sequentially sputtered film Fox/TiO_2_, the light activates the FeOx exposed to the surface. FeOx screens the light before it reaches TiO_2_. Light in a limited spectral region reaching the TiO_2_ can induce ROS. This is the reason why the ROS induced by TiO_2_ did not enhance bacterial cell wall damage.

Leyland *et al.*^37^ recently reported that during photocatalytic bacterial inactivation, a contribution of H_2_O_2_ from bacterial cells respiration is possible. It is possible that the H_2_O_2_ lead to a photo-Fenton type bacterial inactivation on the co-sputtered (FeOx-TiO_2_) or the sequentially sputtered (FeOx/TiO_2_) samples. This would be a minor contribution to the ROS generated on the FeOx photocatalyst. Fagan *et al.*^38^ recently reported details of the ROS evolution on TiO_2_ under visible light irradiation. In the present study we believe that the H_2_O_2_ produced by the ROS interacts with H_2_O or OH^−^ absorbed on the catalyst surface reacting with OH° to form the HO_2_°/O_2_°^−^ leading to bacterial inactivation^39^.

### Surface roughness and film *redox* properties determined by XPS-studies

**Figure 7:**
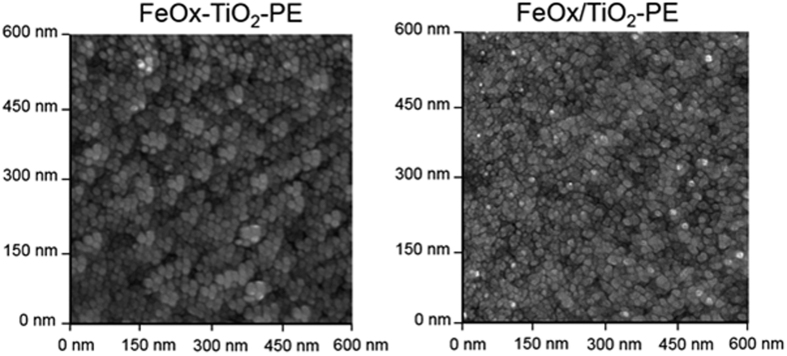
AFM imaging of sequentially sputtered FeOx/TiO_2_-PE with a roughness of 11 nm and co-sputtered FeOx-TiO_2_-PE with a roughness of 24 nm.

Figure 7 shows the roughness (Rg) of the co-sputtered FeO_X_-TiO_2_-PE films was ~24 nm compared with a value of ~11 nm found for the sequentially sputtered FeOx/TiO_2_-PE due to the difference in the coating thicknesses found for both films. The co-sputtered films in Fig. 7a show FeOx nano-particle sizes of 15–35 nm and of 10–15 nm for the TiO_2_ nano-particles. The particle size and surface diffusion controls the mass transport and the particle-growth determining the surface roughness^33^. Sequentially sputtered films on PE alone show FeOx sizes of 20–40 nm on the topmost layers. In the sequentially sputtered films the FeOx ad-atom motions lead to agglomerates with a different size through collective diffusion^34^.

**Figure 8:**
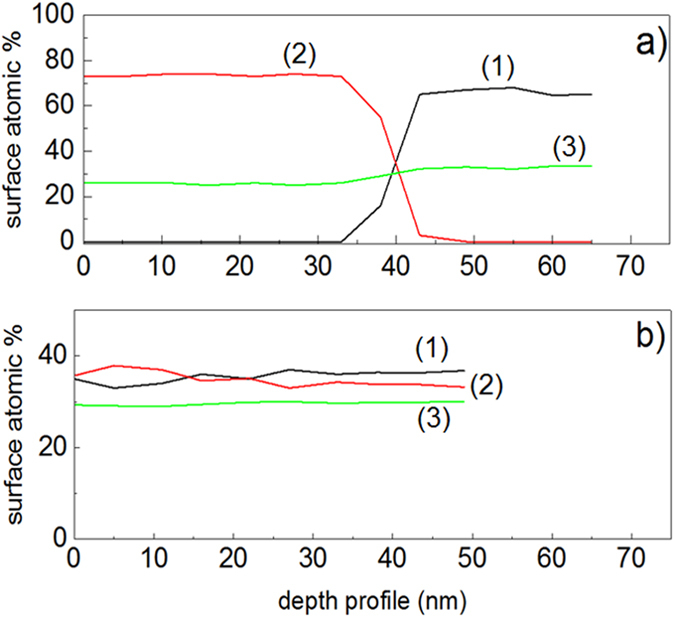
(**a**) XPS etching by way of a beam of 5 kV Ar-ion for: (**a**) sequentially sputtered FeOx/TiO_2_-PE film (1) Ti2p, (2) Fe2p and (3) O1s and (**b**) co-sputtered FeOx-TiO_2_-PE film showing the atomic percentage concentration of atoms (1) Ti2p, (2) Fe2p and (3) O1s in the topmost layers (2 nm) as a function of penetration depth.

Figure 8a presents the X-ray photoelectron spectroscopy (XPS) for sequentially sputtered films. Figure 8 reports the atomic percentage composition as a function of the etching depth for Fe, Ti and O. The etching of the film surface was carried out by Ar-ions of 5 kV reaching a depth of ~50 nm (~250 layers). The TiO_2_ under-layers are seen in Fig. 8 after ~30 nm and reach 60% atomic concentration >45 nm. The O-enrichment was seen to keep stable at 25–30% up-to 65 nm. Figure 8b shows that the Ti and Fe layers were similar within 50 nm (250 atomic layers). The O-enrichment of the TiO_2_ and FeOx kept steady around 30%.

**Figure 9:**
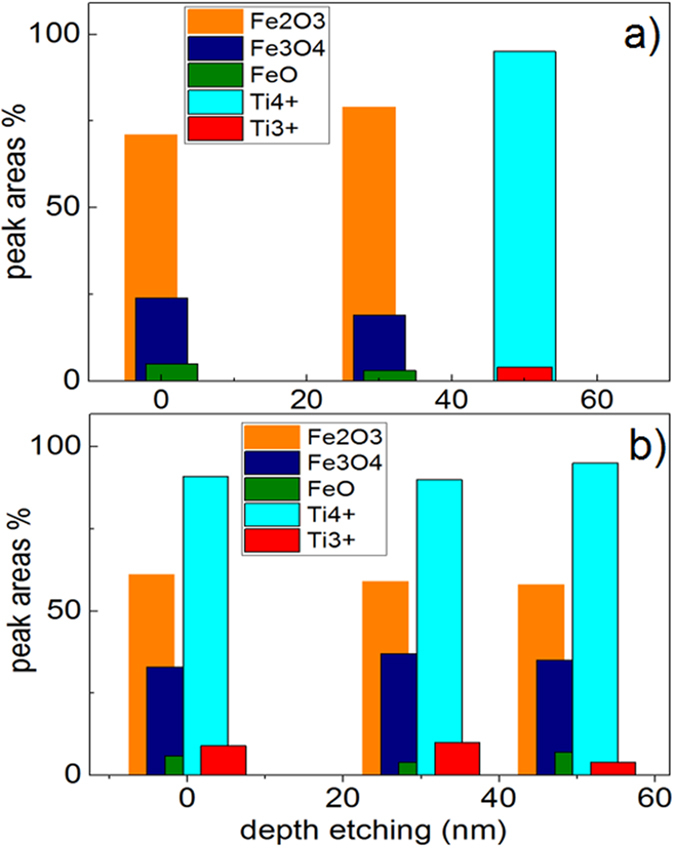
Ti2p and Fe2p oxidation-states evolution during bacterial inactivation as a function of disinfection time as determined by XPS for: (**a**) sequential sputtered FeOx/TiO_2_-PE film and (**b**) co-sputtered FeOx-TiO_2_-PE film. Irradiation source Suntest simulated (52 mW/cm^2^) in the presence of a cut-off filter 400 nm.

Figure 9a presents the changes in the Fe-oxidation states for sequentially sputtered FeOx/TiO_2_-PE films within the 60 min. The initial Fe_2_O_3_ was seen to increase from ~70% at time zero to ~80% after 30 min at the expense of Fe_3_O_4_ and FeO. Figure 9a also shows that after 60 min, the Fe_2_O_3_ reaches ~90%. After 45 min the Ar-etching reaches the depth necessary to deplete the Fe-oxide layers as shown previously in Fig. 8a. The positions of the Fe-oxide peaks used to identify the shift of the oxides followed the values reported in refs 40, 41, 42. Figure 9b shows the changes for the FeOx and TiO_2_ oxidation states for the oxides in the co-sputtered films within the disinfection time. The initial 60% Fe_2_O_3_ percentage remained constant during the disinfection time. Figure 9b shows that the Fe_3_O_4_ and FeO percentages were conserved up-to 60 min. The TiO_2_ (Ti^4+^) slightly increased with a concomitant decrease of the Ti^3+^-oxidation state as shown in Fig. 9b.

## Conclusions

The co-sputtered FeO_X_-TiO_2_-PE films led to a faster bacterial inactivation kinetics compared to the sequentially sputtered films due to a decrease in the rate of the FeOx electron-hole recombination rate due to e-injection from FeOx into low lying TiO_2_ trapping sites. The hetero-junction between FeOx and TiO_2_ seems to promote the directional electron flow in the co-sputtered films leading to a faster of bacterial inactivation. The film composition, optical properties, particle size, film roughness and *redox* states are reported for both films. The results obtained by scavenging experiments indicated that the co-sputtered films were induced in a bigger amount of highly oxidative radicals leading to bacterial inactivation compared to the sequentially sputtered films. The redox reactions during bacterial inactivation were accounted by XPS. The sequentially sputtered films lead to a lower contact angle (CA) reduction compared to co-sputtered films under light irradiation. The co-sputtered FeOx-TiO_2_-PE films due to their faster bacterial inactivation kinetics show a potential to improve the removal of pathogens and prevent biofilm formation under sun/visible light.

## Methods

### Sputtering details, Content and Optical Films Properties

The FeOx was sputtered from a 5 cm diameter target (Kurt Lesker, East Sussex, UK) by reactive direct current magnetron sputtering (DCMS) on PE positioned at 10 cm. Since Fe is paramagnetic, the target was modified creating an eroded area in order to let the magnetic field pass inducing a hopping trajectory along the target. In the case of the FeO_X_-TiO_2_-PE samples, TiO_2_ was co-sputtered simultaneously with Fe from 5 cm targets (Kurt Lesker, East Sussex, UK) by reactive direct current magnetron sputtering (DCMS). The current intensity was adjusted to attain the desired film deposition rate of Fe and Ti in the Ar + O_2_ gas atmosphere. The sequential sputtering of the FeOx/TiO_2_-PE films was carried out under similar experimental conditions.

The Fe- and Ti-content of sputtered on PE film were evaluated by X-ray fluorescence (XRF) in a PANalytical PW2400 spectrometer. The XRF analysis detects the content of the Fe/Ti layers on PE and the results are shown in Table 2.

Diffuse reflectance spectroscopy (DRS) was carried out in a Perkin Elmer Lambda 900 UV-VIS-NIR spectrometer provided for with a PELA-1000 accessory within the range of 200–800 nm and a resolution of 1 nm. The polyethylene (PE) film used consisted of a highly branched low crystalline semi-transparent film with the formula H(CH_2_-CH_2_)H. The (LDPE) 0.1 mm thick was obtained from Goodfellow, UK had a density of 0.92 g/cm^3^.

### Femtosecond Spectroscopy of Films

The output of a Ti sapphire oscillator (800 nm, 80 MHz, 80 fs, «Tsunami», «Spectra-Physics», USA) was amplified by a regenerative amplifier system («Spitfire», «Spectra-Physics», USA) at the repetition rate of 1 KHz. The amplified pulses were split into two beams. One of the beams was directed into a non-linear phase-matched optical amplifier with the output centred at 710 nm compressed by a pair of quartz prisms. The Gauss pulse was tuned at 40 fs/425 nm. The second beam was focused onto a thin quartz cell with H_2_O to generate super-continuum probe pulses. The probe pulses were time-delayed with respect to each. The pulses were then attenuated, recombined, and focused onto the sample cell. The pump and probe light spots had diameters of 300 and 120 μm, respectively. The pump pulse energy was attenuated to 65 nJ in some cases to optimize the acquisition of data. The experiments were carried out at 278 K. Laser pulse frequency was adjusted by a control amplifier SDG II Spitfire 9132, manufactured by Spectraphysics (USA). The pulse operation frequency was 50 Hz, which is sufficiently low to exclude permanent bleaching of the sample.

The circulation rate in the flow cell was fast enough to avoid multiple excitations in the sample volume. The relative polarizations of pump and probe beams were adjusted to 54.7° (magic angle) in parallel and perpendicular polarizations. The super continuum signal out of the sample was dispersed by a polychromator («Acton SP-300») and detected by CCD camera («Roper Scientific SPEC-10»). Transient absorption spectral changes ∆A (t, λ) were recorded within the range of 380–800 nm. Because the super-continuum is chirped, a time correction was applied at each kinetic trace. Control experiments were carried out for non-resonant signals of coherent spike from net PE films.

### Bacterial Inactivation Kinetics and Oxidative Intermediate Radicals

The samples of *Escherichia coli* (*E. coli K12* ATCC23716) on 2 α by 2 cm FeOx-TiO_2_ PE were placed into a glass Petri dish and irradiated in the cavity reactor. The irradiation of the samples was carried out in a cavity of a sunlight simulator with a light dose of 52 mW/cm^2^ provided for with a filter blocking the light <400 nm. Other details for the CFU counting within the disinfection time have been recently reported^15^. The CFU statistical analysis of the bacteria inactivation data was performed taking into account the standard deviation. 1,4-benzoquinone (BQ) and methanol were used respectively as O_2_°^−^ radical scavenger and OH° scavengers^11^,^14^. Ethylenediamine tetra-acetic acid (EDTA-2Na) was used as TiO_2_ vb(h^+^) scavenger^15^.

### Characterization of the surface properties co-sputtered and sequentially sputtered FeOx-TiO_2_ films

Film roughness (Rg) and particle size were determined by atomic force microscopy. The AFM images were acquired in contact mode using a PSIA Xe-100 AFM. Silicon nitride cantilevers were used with feedback set points around 1.0 nN. The images originate from the Z-scanner and are not influenced by the non-linearity and the hysteresis of the z-scanner. The AFM scanner and position sensors were calibrated using standard samples from Mikromash. The experimental error in the roughness was below 10% for the selected 600 nm × 600 nm scanned area. The mean surface roughness (Rg) was calculated for the scanned area. The mean surface roughness values (Rg) involve an experimental error below 10%. The wettability of the PE-TiO_2_ films was determined by the water droplet contact angle (CA) by the sessile drop method on a DataPhysics OCA 35 unit. Drop volumes of 0.5 microliter were chosen in all experiments to avoid shape alteration due to gravitational forces and to diminish the evaporation effects. The measurements were performed at room temperature (65% controlled humidity). The drop image was registered in a CCD camera (1280 × 960 pixels) attached to a microscope and processed by way of software image analysis.

The X-ray photoelectron spectroscopy (XPS) of the Cu-Ag films was determined using an AXIS NOVA photoelectron spectrometer (Kratos Analytical, Manchester, UK) provided for with monochromatic AlK_a_ (hn = 1486.6 eV) anode. The carbon C1s line with position at 284.6 eV was used as a reference to correct the charging effects. The surface atomic concentration was determined by XPS from the peak areas, using the known sensitivity factors for Ti, Fe and O. The surface percentages composition was determined within 10 atomic layers (2 nm)^40^,^41^. Spectrum background was subtracted according to Shirley^42^. The depth profile determination took several minutes. The PE penetration rate was referenced with the known rate of tantalum (Ta) of 15 atomic layer/min or around 3 nm/min^42^.

## Additional Information

**How to cite this article**: Rtimi, S. *et al.* FeOx-TiO_2_ Film with Different Microstructures Leading to Femtosecond Transients with Different Properties: Biological Implications under Visible Light. *Sci. Rep.* **6**, 30113; doi: 10.1038/srep30113 (2016).

## Supplementary Material

Supplementary Information

## Figures and Tables

**Table 1:** Kinetic constants for femto-picosecond decay in FeOx/TiO_2_-PE and co-sputtered FeOx-TiO_2_-PE obtained by Principal Component analysis.

	FeOx/TiO_2_-PE		FeOx-TiO_2_-PE	
	Constant (1/ps)	Amplitude of fitting	Constant (1/ps)	Amplitude of fitting
PC1	0.20 ± 0.011	0.033 ± 0.001	0.0054 ± 0.001	−0.02 ± 0.01
	2.2 ± 0.13	0.05 ± 0.002	0.13 ± 0.006	−0.04 ± 0.02
			2.6 ± 0.0004	−0.057 ± 0.021
PC2	11 ± 2	0.023 ± 0.006	21 ± 1	−0.068 ± 0.008
PC3	21 ± 1	−0.2 ± 0.02	0.39 ± 0.1	0.002 ± 0.0003
			18 ± 3	0.06 ± 0.03

**Table 2:** Fe and Ti-loadings of FeOx-TiO_2_-PE films detected by X-ray fluorescence (XRF).

	Species	Wt%/wt PE (error% = 0.006)
FeOx-TiO_2_-PE co-sputtered for 2 min	FeOx	0.045
	TiO_2_	0.033
FeOx/TiO_2_-PE sequentially sputtered for 8 min TiO_2_ followed by 2 min FeOx	FeOx	0.08
	TiO_2_	0.16
FeOx (sputtered for 2 min)	FeOx	0.09
TiO_2_ (sputtered for 8 min)	TiO_2_	0.18
