# Supplementary material for: FeOx-TiO2 Film with Different Microstructures Leading to Femtosecond Transients with Different Properties: Biological Implications under Visible Light
Source: Sci Rep. 2016 Jul 22;6:30113. doi: 10.1038/srep30113 (PMC4957123; doi:10.1038/srep30113)
Supplement: Supplementary Information [file srep30113-s1.pdf]

$FeOx-TiO_2$ Film with Different Microstructures Leading to Femtosecond Transients with Different Properties: Biological Implications under Visible Light

Sami Rtimi[1]*, Cesar Pulgarin[1], Victor A. Nadtochenko[2,3], Fedor E. Gostev[2], Ivan V. Shelaev[2], John Kiwi[1]

[1] Ecole Polytechnique Fédérale de Lausanne, EPFL-SB-ISIC-GPAO, Station 6, CH-1015 Lausanne, Switzerland.
[2] N. N. Semenov Institute of Chemical Physics, Russian Academy of Sciences, str. Kosygina 4, 119991 Moscow, Russia.
[3] Moscow State University, Department of Chemistry, Leninskiye Gory 1-3, 119991, Moscow, Russian Federation.

Supplementary Material S1,

Fitting the decay $FeOx-TiO_2$-Fe transients

Probe wavelength for $FeOx-TiO_2$-PE are: 1 (green) 610 nm; 2 (red) 735 nm

Exponential fit parameters of y0+A1 exp(- constant1 * t) +A2 exp(- constant2 * t) are:

For 1 (green), 610 nm: y0= 0.0005 ± 6.110^{-5}, A1 = 0.0011 ± 4.2 10^{-5}, constant1 = 0.004 ± 0.5 1/ps; A2 = 0.002 ± 1 10^{-4}; constant2= 0.11 ± 0.009 1/ps

For 2 2 (red) 735 nm y0 = 9. 10^{-5}± 7 10^{-5}; A1 = 0.0006 ± 5. 10^{-5}; constant1 = 0.003 ± 0.0007 /ps; A2 = 0.0009 ± 6 10^{-5} ; constant2 = 0.09 ± 0.001 1/ps

Insert. Probe wavelength is: 1 (green) 610 nm; 2 (red) 735 nm; 3 (blue) 465 nm red

Exponential fit parameters of y0+A exp(- constant * t) are :

For 1 (green), 610 nm: y0= 0.003 ± 1.5 10^{-5}; A= 0.0034 ± 4.310^{-5}; constant = 2.4 ± 0.06 1/ps

For 2 2 (red) 735 nm y0 = 0.002 ± 1.4 10^{-5}, A= 0.003 ± 7.2 10^{-5}, constant = 3.5 ± 0.13

Supplementary Material S2, Fitting the decay FeOx-TiO_2-Fe transients

Probe wavelength for FeOx/TiO_2-PE are: 1 (green) 560 nm; 2 (red) 690 nm

Exponential fit parameters of y0+A1 exp(- constant1 * t) +A2 exp(- constant2 * t) are:

For 1 (green), 560 nm: y0= 0.0008 ± 1.4 10^{-5}, A1 = 0.0002 ± 1.7 10^{-5}, constant1 = 0.004 ± 0.5 1/ps; A2 = 0.0019 ± 7.3 10^{-5}; constant2= 0.2 ± 0.01 /ps

For 2 2 (red) 690 nm

y0+A exp(- constant * t)

y0 = -0.00038 ± 5.2 10^{-6}; A = 0.0009 ± 5.0 10^{-5}; constant1= 0.23 ± 0.019 1/ps;

Insert. Probe wavelength is: 1 (green) 610 nm; 2 (red) 690 nm;

Exponential fit parameters of y0+A exp(- constant * t) are :

For 1 (green), 560 nm: y0= 0.0021 ± 1.6 10^{-5}; A= 0.0028 ± 4.6$10^{-5}$; constant = 1.7 ± 0.051/ps

For 2 2 (red) 690 nm y0 = 7.9 10^{-5} ± 2.2 10^{-5}, A= 0.0016 ± 3 10^{-5}, constant = 1.3 ± 0.07 1/ps

Supplementary Material S3

SVD decomposition of the experimental matrix data obtained for both films whose absorption up to 100ps is shown in Figure 3 indicate that significant singular values for the FeOx-TiO_2-PE and FeOx/TiO_2-PE film decay are about the same and not more than 4.

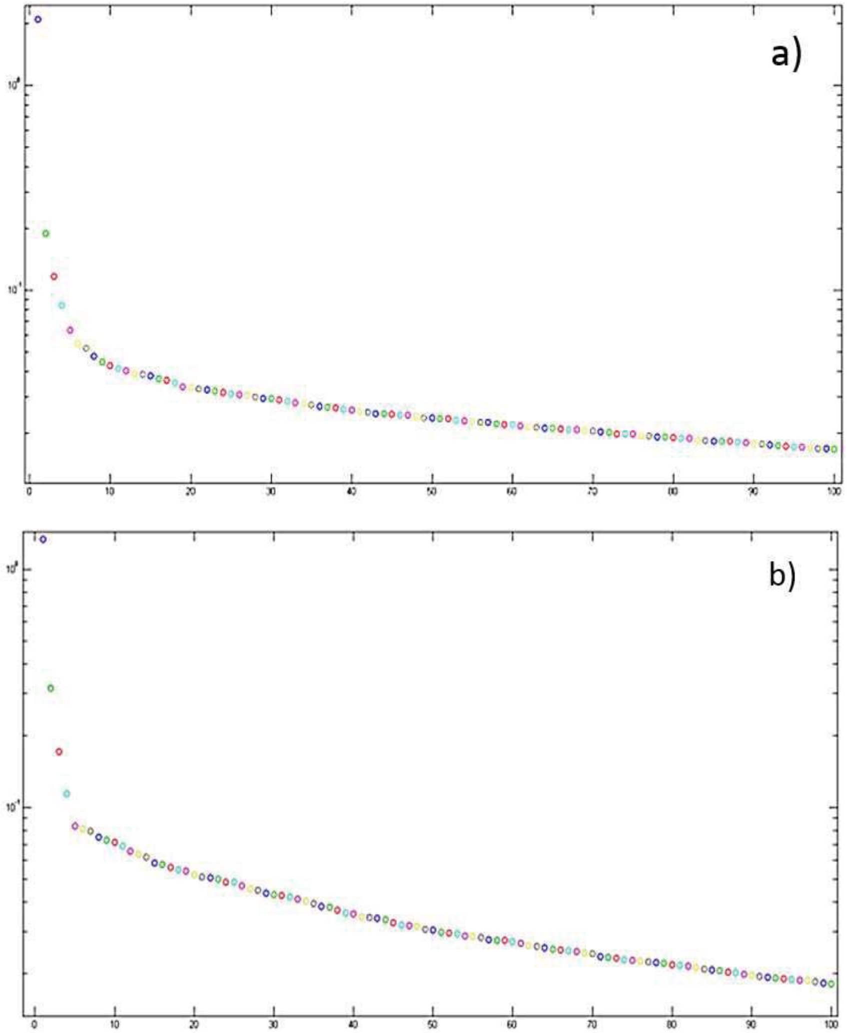

S3. First 100 singular values: Diagonal elements taken for S. a) FeOx-TiO_2-PE, b) FeOx/TiO_2-PE.

Supplementary Material S4

The main information obtained by SVD is the rank of the data matrix analyzed, i.e., the number of components required to describe the measured process. This information enables the determination of the number of absorbing species in the reaction on a completely model-free basis. Singular value decomposition of the matrix **D=delta Absorbance** (λ,t) is usually written as in Eq. (1),

$D(\lambda,t) = U(\lambda)\ S\ V^T(t)$ (1)

Where: **U** and **V** are orthonormal matrices of dimensions m × n and n × n, respectively. The matrix **S** is diagonal and contains the singular values arranged in descending order. The number of components can be estimated from the number of significant singular values, i.e., values clearly higher than noise-related one. Singular value decomposition provides a means to describe the data into orthogonal vectors, i.e., linearly independent information, and in this sense, helps to determine the minimum number of intermediates needed. For more extensive in formation see G.H. Golub, C.F. Van Loan, Matrix Computation, Second Edition, The John Hopkins University Press, London, 1989.
